# A Multivariate Pattern Analysis of Metabolic Profile in Neurologically Impaired Children and Adolescents

**DOI:** 10.3390/children8030186

**Published:** 2021-03-01

**Authors:** Valeria Calcaterra, Giacomo Biganzoli, Gloria Pelizzo, Hellas Cena, Alessandra Rizzuto, Francesca Penagini, Elvira Verduci, Alessandra Bosetti, Daniela Lucini, Elia Biganzoli, Gian Vincenzo Zuccotti

**Affiliations:** 1 Pediatric and Adolescent Unit, Department of Internal Medicine, University of Pavia, 27100 Pavia, Italy; 2 Pediatric Department, “V. Buzzi” Children’s Hospital, 20154 Milan, Italy; francesca.penagini@asst-fbf-sacco.it (F.P.); alessandra.bosetti@asst-fbf-sacco.it (A.B.); 3 Pharmacogenomics & Precision Therapeutics Master Degree, University of Milan, 20142 Milan, Italy; alessandrastefania.rizzuto@studenti.unimi.it; 4 Pediatric Surgery Unit, “V. Buzzi” Children’s Hospital, University of Milan, 20154 Milan, Italy; gloriapelizzo@gmail.com; 5 Department of Biomedical and Clinical Science “L. Sacco”, University of Milan, 20157 Milan, Italy; gianvincenzo.zuccotti@unimi.it; 6 Laboratory of Dietetics and Clinical Nutrition, Department of Public Health, Experimental and Forensic Medicine, University of Pavia, 27100 Pavia, Italy; hellas.cena@unipv.it; 7 Clinical Nutrition and Dietetics Service, Unit of Internal Medicine and Endocrinology, ICS Maugeri IRCCS, 27100 Pavia, Italy; 8 Department of Health Sciences, University of Milan, 20146 Milan, Italy; elvira.verduci@unimi.it; 9 Department of Medical Biotechnologies and Translational Medicine, University of Milan, 20133 Milan, Italy; daniela.lucini@unimi.it; 10 Department of Clinical Sciences and Community Health & DSRC, University of Milan, 20122 Milan, Italy; elia.biganzoli@unimi.it

**Keywords:** disability, children, metabolic syndrome, insulin resistance

## Abstract

Background: The prevalence of pediatric metabolic syndrome is usually closely linked to overweight and obesity; however, this condition has also been described in children with disabilities. We performed a multivariate pattern analysis of metabolic profiles in neurologically impaired children and adolescents in order to reveal patterns and crucial biomarkers among highly interrelated variables. Patients and methods: We retrospectively reviewed 44 cases of patients (25M/19F, mean age 12.9 ± 8.0) with severe disabilities. Clinical and anthropometric parameters, body composition, blood pressure, and metabolic and endocrinological assessment (fasting blood glucose, insulin, total cholesterol, high-density lipoprotein cholesterol, triglycerides, glutamic oxaloacetic transaminase, glutamate pyruvate transaminase, gamma-glutamyl transpeptidase) were recorded in all patients. As a control group, we evaluated 120 healthy children and adolescents (61M/59F, mean age 12.9 ± 2.7). Results: In the univariate analysis, the children-with-disabilities group showed a more dispersed distribution, thus with higher variability of the features related to glucose metabolism and insulin resistance (IR) compared to the healthy controls. The principal component (PC1), which emerged from the PC analysis conducted on the merged dataset and characterized by these variables, was crucial in describing the differences between the children-with-disabilities group and controls. Conclusion: Children and adolescents with disabilities displayed a different metabolic profile compared to controls. Metabolic syndrome (MetS), particularly glucose metabolism and IR, is a crucial point to consider in the treatment and care of this fragile pediatric population. Early detection of the interrelated variables and intervention on these modifiable risk factors for metabolic disturbances play a central role in pediatric health and life expectancy in patients with a severe disability.

## 1. Introduction

Metabolic syndrome (MetS) refers to a clustering of co-incident and interrelated risk factors that place a subject at high risk of developing cardiovascular disease (CVD) and diabetes type 2 (DT2), thus increasing mortality risk [1,2,3,4,5,6,7,8,9]. These factors include central obesity, dysglycemia/insulin resistance (IR), hypertension, high levels of triglycerides (TG), and low high-density lipoprotein cholesterol (HDL cholesterol) [1,2,3,4,5,6,7,8,9].

The prevalence of pediatric MetS ranges from 0.3 to 26.4%. This variation in prevalence is due to the large number of different pediatric definitions of MetS [9]. MetS is usually closely linked to overweight and obesity, and it predicts DT2, CVD, and all-cause mortality in adults [1,2,3,4,5,6,7,8,9]. However, this condition has also been described in subjects of normal weight [6,8,10,11,12,13,14], undernourished children with disabilities [15,16], or older non-obese adults, particularly when affected by sarcopenia [17,18,19]. The data in non-obese subjects with disabilities, who are considered to have MetS if they meet more than three pathological metabolic parameters, such as glucose metabolic disorders and/or dyslipidemia, and/or hypertension [15], suggest the crucial role that body composition plays, particularly loss of lean mass, in the risk of developing MetS [15,16]. In these subjects, multifactorial pathogenesis of MetS has been proposed, including malnutrition, the assumption of drugs, chronic inflammation, oxidative stress, high cumulative biological dysregulation, and restricted physical activity [16,17,18,19,20,21].

Considering the improvement in survival rates of patients with disabilities over the past half a century [22,23,24], the care and management of this group are of particular importance in order to improve comorbid metabolic issues, to promote their chances of growth and survival, and to prevent premature mortality.

Multivariate pattern analysis is a widely applied biomedical research tool used in the treatment and diagnosis of diseases [25,26] for its ability to take into account the structure of correlation and explore relevant associations within the data.

The aim of this study was to perform a multivariate pattern analysis of the metabolic profile in neurologically impaired children and adolescents in order to reveal patterns and crucial biomarkers among highly interrelated variables. Early detection of metabolic disorders may be crucial for the treatment and care of patients with a severe disability.

## 2. Patients and Methods

### 2.1. Patients

We retrospectively considered 44 patients (25 males, 19 females, mean age 12.9 ± 8.0 yrs standard deviation (SD)) with severe disabilities (Level 5 according to the Gross Motor Function Classification System) [27] who were bedridden and lived at home or in sheltered communities. In all patients, body mass index (BMI) < 2SD was calculated as previously described [15,16]. The patients had been referred to our Pediatric Endocrinological Unit for auxological evaluation or to the Pediatric Surgical Unit for treatment and/or management of nutrition support. Diagnoses included cerebral palsy due to hypoxic-ischemic damage (38.6%), dysmorphic syndrome (34.1%), and epileptic encephalopathy (27.3%). In 42/44 of the patients (95.4%), anticonvulsive drugs were administered (at least two of the following: phenobarbital, phenytoin, valproic acid, topiramate, lamotrigine, carbamazepine, and clonazepam). Bolus (72.7%) or continuous (27.3%) enteral feeding was performed in all subjects.

Clinical and anthropometric parameters, body composition, and biochemical and endocrinological assessments were recorded in all patients.

As a control group, we evaluated 120 healthy Caucasian children and adolescents comparable for age and sex (61 males, 59 females, mean age 12.9 ± 2.7 years SD), who were enrolled in our center as controls for other metabolic studies. All the parents or guardians provided their consent to retrospectively enroll the subjects in other studies for clinical research purposes, epidemiology, study of pathologies, and training aimed at improving knowledge, care, and prevention. The study was performed according to the Declaration of Helsinki and with the approval of the Institutional Review Board.

### 2.2. Methods

#### 2.2.1. Anthropometric Parameters and Body Composition

Physical examination of the participants included evaluation of weight, height and body segment, lengths according to Stevenson’s method, waist circumference, BMI, pubertal stage according to Marshall and Tanner [28,29], and blood pressure (BP) measurements, as previously detailed [15,16].

#### 2.2.2. Biochemical and Endocrinological Parameters

Blood samples were drawn in the morning after overnight fasting. Metabolic and hormonal blood assays included fasting blood glucose (FBG), insulin, total cholesterol, high-density lipoprotein (HDL) cholesterol, tryglicerides (TG), glutamic oxaloacetic transaminase (GOT), glutamate pyruvate transaminase (GPT), and gamma-glutamyl transpeptidase (GGT). As previously described [15,16], plasma glucose was measured using the hexokinase-G-6-phosphate dehydrogenase method (Siemens Healthcare Diagnostics, Camberley, UK) with a chemistry analyzer (Advia XPT, Siemens). An enzymatic method (Advia XPT, Siemens Healthcare Diagnostics, U.K.) was used to determine total cholesterol. HDL cholesterol was measured by means of the selective detergent method, followed by enzymatic reactions (Siemens Healthcare Diagnostics). The glycerol phosphatase oxidase method was used to measure TG concentrations (Siemens Healthcare Diagnostics, U.K.). Serum insulin was measured with a solid-phase, two-site chemiluminescent immunometric assay with an immunochemistry analyzer (Immulite 2000, Siemens Healthcare Diagnostics, U.K.). AST, ALT, and GGT were measured with a chemistry analyzer (Advia XPT, Siemens Healthcare) equipped with dedicated reagents; the transaminase assay method is based on nicotinamide adenine dinucleotide ((NAD)H monitoring by ultraviolet (UV) detection without the addition of P-5′-P. The GGT assay method is based on the transfer of the gamma-glutamyl group from L-gamma-glutamyl-3-carboxy-4-nitroaniline to the glycylglycine acceptor to yield 3-carboxy-4-nitroaniline, which is measured.

Insulin resistance was determined by means of the homeostasis model assessment for insulin resistance (HOMA-IR) using the following formula: insulin resistance = (insulin × glucose)/22.5 [30].

The trygliceride glucose index (TyG index) was calculated as the Ln[fasting triglycerides (mg/dL)×fasting plasma glucose (mg/dL)/2] as a surrogate marker of IR and predictor of diabetes [31].

## 3. Statistical Analysis

In order to explore the anthropometric and metabolic characteristics of the two groups (children with disabilities and healthy controls), a univariate statistical analysis was performed. To estimate the probability density function of the variables analyzed, a non-parametric kernel density estimation method was applied, representing function by means of violin plots, including a scatterplot and a bar chart showing mean and standard deviation values, in order to obtain a comprehensive view of the distribution. The graphs were grouped by sex. This method allowed us to identify patterns in the distributions of the variables. An examination of the summary statistics and boxplots would not highlight these patterns sufficiently.

The analyses were conducted separately in the two groups at first and subsequently in a merged dataset for the variables that the two groups shared. To measure the linear correlation between each pair of variables, Pearson correlation coefficients were computed and shown in a matrix correlation plot.

To study the potential association between the studied parameters, a principal component analysis was performed. This method allowed us to reduce the dimensionality of the dataset by projecting each data point onto the first few principal components (up to three) while preserving as much of the data variation as possible. This method consists of rotating the axes of the multivariate space of the original variables, along orthogonal directions of maximal variance (principal components PCs), and creating a new space defined by the PCs. Each of the PCs is characterized by a percentage of explained variance of the data. If a relevant amount of variance is explained by the first PCs, the projection of the variables as vectors on the subspace defined by the new axes can be useful in exploring correlation structures present in the data. If two variables are strongly correlated, they are projected close together (the correlation is positive) or conversely with maximum alignment (the correlation is negative). Otherwise, if they do not display correlation, they tend to be projected at an angle of 90 degrees. Moreover, if the subspace defined by the two principal components describes the variables well, these are projected toward a circle of radius unity, also known as the circle of correlation; otherwise, they tend to be projected close to the origin of the axes. Only the variables common to both groups were considered in the analysis.

Finally, to assess the association between the condition of disability and the anthropometric and metabolic variables accounting for their associations, a proportional odds multivariable logistic regression model using maximum likelihood estimation was employed. Non-linear effects were taken into account by modeling the restricted cubic splines (RCS) transformation for the variables. Restricted cubic splines (RCS) with 3 knots and 2 degrees of freedom transformation were considered. The statistical significance of the coefficients was tested by combined Wald ANOVA tests, both for linear and non-linear effects. A stepwise variable selection method considering the Akaike Information Criterion (AIC) was used to assess the chance of reducing model complexity in favor of its interpretability. The coefficients resulting from the final model indicated the relative change in the log of the odds of being a child with disabilities (1) compared to being a healthy control child (0) for a unit change of the corresponding model predictor. Graphs showing the conditional effects of the variables were generated by setting the values of the adjusting variables at their medians. Furthermore, in order to test whether the regression parameters were affected by the presence of possible outliers, a robust model was fitted by using the glmRob function of the R package robustbase V093-6. All the statistical analyses were conducted with R software (version 4.0.0).

## 4. Results

### 4.1. Univariate Analysis

In Table 1, the clinical features of the patients and controls are reported. The groups were comparable for pubertal stages.

For the children-with-disabilities group, 44 observations were available, and they showed missing values in some of the variables (HDL cholesterol = 1, systolic BP = 6, diastolic BP = 6). For the healthy control group of children, 120 observations were available and also in this case they showed missing values in some of the variables (fasting insulin = 4, HOMA-IR = 4, HDL cholesterol = 10, total cholesterol = 10, systolic BP = 1, diastolic BP = 1). Table 1 summarizes the main statistics of the variables considered in the univariate analysis for both groups of patients. Major differences between the two groups are evident for fasting insulin, HOMA-IR, fasting triglycerides, and HDL cholesterol.

**Figure 1:**
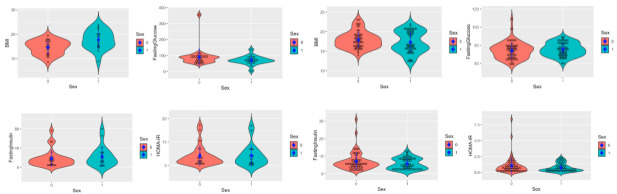

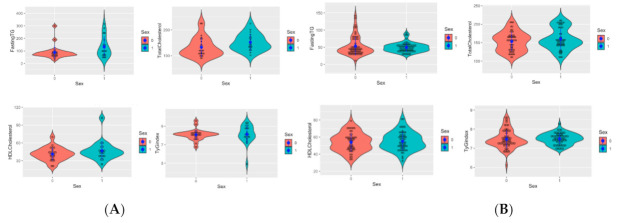
The panel shows the kernel density estimation by means of violin plots for the variables related to the metabolism of the children-with-disabilities group (**A**) and the healthy control group (**B**). In red, the distribution of observations in females, and in blue, the distribution of observations in males.

Figure 1 reports the distribution of the variables under examination in the two groups: children with disabilities (panel A) and healthy control children (panel B). The violin plots, including the scatterplot, show the differences between the distribution of the variables in males and females. Overall, the children-with-disabilities group showed a more widely dispersed distribution of the variables related to glucose metabolism and insulin resistance compared to the healthy controls. Specifically, there were differences between the disabled children group and the healthy control children in the biochemical variables of fasting insulin, HOMA-IR, fasting triglycerides, and HDL cholesterol, but there were no major differences between the two sexes within either group.

Concerning the children-with-disabilities group, major differences in dispersion were found in fasting TG and the TyG index, with males showing a wider distribution compared to females. In the healthy control children, females displayed more clustered observations for BMI compared to males, whereas the distribution of fasting TG and the TyG index was more dispersed in females than in males.

### 4.2. Multivariate Analysis

The first step of the multivariate analysis consisted of evaluating the correlation among the variables by computing the Pearson correlation coefficients for every pairwise association after normalization of the variables for their standard deviation.

The coefficients were computed separately for the two groups and subsequently in the variables of the merged group. The results were represented in matrix correlation plots.

Since pairwise associations do not take into account the joint relationships with the other variables, a thorough investigation of the association structure among the parameters was performed by exploiting the principal component analysis (PCA).

**Figure 2:**
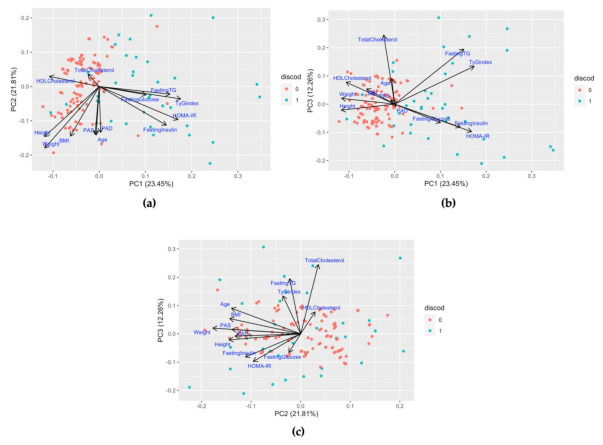
The panel shows biplot graphs in which the observations of the merged dataset are scattered in a two-dimensional plane defined by the PCs. The projections of the variables under examination in the analysis are shown as black arrows and are labeled. The observations derived from the healthy control group are in red, whereas those of the children-with-disabilities group are in blue. It is clear that when considering the plane defined by the PC1 and PC2 (**a**) or by the PC1 and PC3 (**b**), we note that the observations from the healthy control children group cluster together in a region of the plane where the PC1 is negative, whereas observations relating to the children with disabilities group are more dispersed in the direction of the projection of the variables related to insulin resistance and glucose metabolism. By excluding the PC1 and considering the PC2 and PC3 (**c**), the observations are found to be mixed, highlighting the fact that the PC1, characterized by the variables related to insulin resistance and glucose metabolism, is crucial in differentiating the two groups.

In the case of the children-with-disabilities group, the first three principal components explained the 56% variance in the data (PC1 = 22 %, PC2 = 18 %, PC3 = 16%). The projections of the original variables on the three planes are shown in Figure 2. The first axis (PC1) was mainly characterized by the weight, height, and BMI variables (which are highly correlated according to the BMI formula). The second axis (PC2) was characterized by the fasting insulin, HOMA-IR, and fasting glucose variables. The third axis (PC3) was characterized by the fasting TG, TyG index, and total cholesterol variables. It seemed that variables such as systolic and diastolic BP were not well represented by the three planes defined by the first three PCs. In fact, the vectors of their projections were close to the origin of the axes. In all of the planes defined by the PCs, it appeared that the fasting insulin, fasting glucose, and HOMA-IRvariables were positively correlated and grouped. In the plane defined by PC1 and PC2, these were uncorrelated to the weight, height, and age variables. In the plane defined by PC1 and PC3, the fasting TG and TyG index variables were positively correlated with each other, although neither correlated with the age and weight variables.

In the case of healthy control children, the first three principal components explained the 66% variance in the data (PC1 = 35%, PC2 = 18%, PC3 = 13%). The first axis (PC1) was mainly characterized by the variables relating to BMI, weight, height, age, fasting insulin, HOMA-IR, fasting glucose, systolic BP, and diastolic BP, which were positively correlated and so grouped together. The fasting TG and TyG index variables (strictly correlated by the TyG index formula) characterized the second axis (PC2), whereas the third axis (PC3) was mainly characterized by the total cholesterol and HDL cholesterol variables.

**Figure 3:**
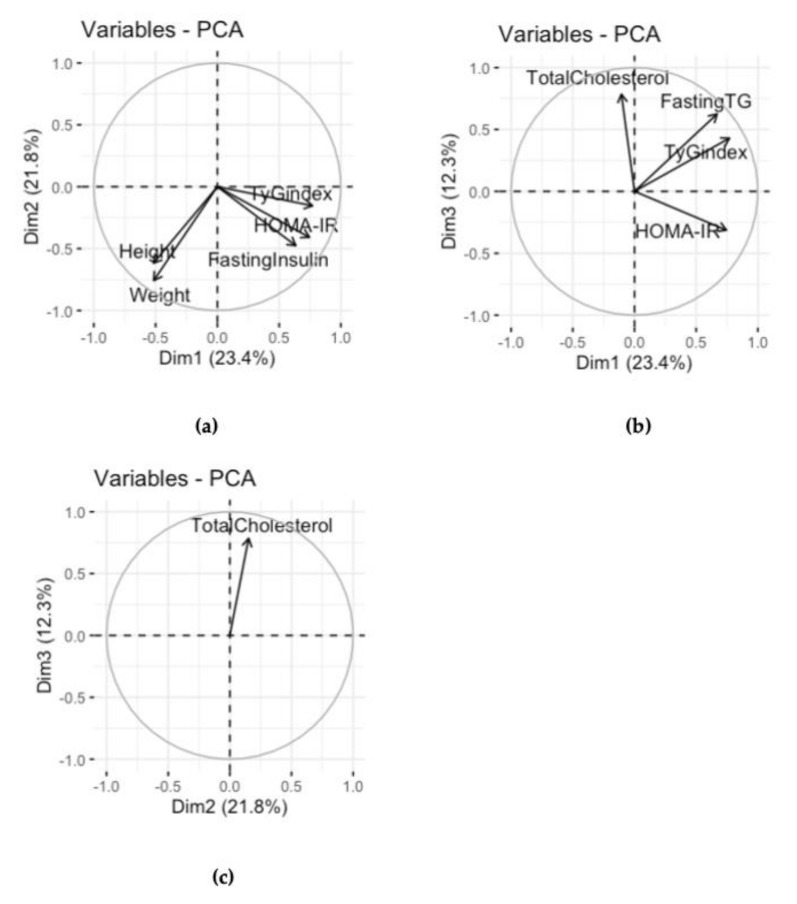
The panel shows the three subspaces defined by the three PCs. When the subspace defined by the two principal components describes the variables well, these are projected toward a circle unity radius, also known as the circle of correlation. As can be seen in (**a**,**b**), the trygliceride glucose index (TyG index), homeostasis model assessment for insulin resistance (HOMA-IR), and fasting insulin variables are all well described by the subspace defined by the PC1 and PC2; they all correlate with each other and also positively correlate with PC1. In (**c**), we can note that the subspace defined by the PC2 and PC3 describes well only the total cholesterol variable.

Finally, the multivariate PCA analysis on the merged group showed that the first three principal components explained the 57% variance in the data (PC1 = 23%, PC2 = 22%, PC3 = 12%). As expected, in this case, whereas the total cholesterol and HDL cholesterol variables represented the third axis (PC3), the height, systolic BP, diastolic BP, weight, BMI, and age variables were all grouped together, and they characterized the second axis (PC2) but not the first (PC1), contrary to what was observed for the healthy control group. Moreover, the first axis (PC1) was mainly characterized by the group of variables related to insulin resistance (fasting glucose, fasting insulin, fasting TG, TyG index, and HOMA-IR). These all positively correlated with each other and positively correlated with the PC1 (Figure 3). In the following phase, we analyzed the three planes defined by the PCs for distribution of all the observations of the merged dataset, stratified and labeled by the presence or absence of disabilities. As expected, in the plane defined by the PC1 with one of the other two dimensions, the observations relating to the healthy control children (absence of disability) were clearly seen to cluster together in the region of the plane where the PC1 is negative, whereas the observations relating to the group of children with disabilities were far more frequently found in the positive values of the PC1. If the PC1 had not been considered, as in the case of the plane defined by the PC2 and PC3, no clusters formed by the observations of the healthy control children group would have been identifiable. Indeed, the observations would have been dispersed as in the case of the children-with-disabilities group, underlining the importance of the PC1 in characterizing the difference between the two groups (Figure 2). Overall, the PC1, which resulted from the principal component analysis conducted on the merged dataset and characterized by the variables related to glucose metabolism and insulin resistance, was crucial in describing the differences between the group of children with disabilities and the group of healthy control children.

### 4.3. Logistic Regression Model

An additive linear multiple logistic regression model was fitted by considering the effects of the predictor variables: age, BMI, weight, HOMA-IR, TyG index, systolic BP, and diastolic BP. Non-linear effects were considered for HOMA-IR and weight, which showed statistical evidence of departure from the linear assumption. These were fitted in a final model in which the variables age, TyG index, and the restricted cubic spline transformation of weight and HOMA-IR were statistically significant (age, *p* = 0.0087; weight, *p* = 0.023 for the linear effects and *p* = 0.017 for the non-linear effects; HOMA-IR, *p* = 0.024 for the linear effects and *p* = 0.011 for the non-linear effects; TyG index, *p* = 0.044), whereas the systolic BP (*p* = 0.047) and diastolic BP (0.10) predictors were included as adjusting variables.

**Figure 4:**
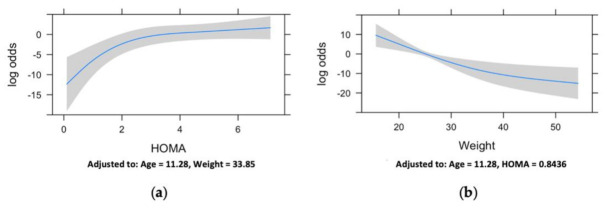
Restricted cubic spline curve showing the model-predicted log odds of being a child with disabilities against homeostasis model assessment for insulin resistance (HOMA-IR) (**a**) and weight (**b**) values. Shadowed areas represent 95% confidence intervals. The curve is derived from a multiple logistic model adjusted for all the covariates. On the Y axis, the conditional effect in terms of the log odds of being a child with disabilities is displayed, whereas, on the X axis, the values of the specific variables are shown. As can be seen in panel (**a**), the HOMA-IR has a major effect in discriminating between the group of children with disabilities and healthy control children at values below 2. In fact, at values greater than 2, the curve almost reaches a plateau. In panel (**b**), the non-linear conditional effect of the weight variable can be observed with a decrease in log odds of being a child with disabilities as weight increases, with a reduction at higher weights.

Concerning the linear effects of age and TyG index, the estimated coefficient yielded positive values according to a proportional increase of the log odds of being disabled with the increase of these variables. As regards the non-linear effects, Figure 4 provides additional details. Namely, the HOMA-IR showed an increasing effect on the log odds of being disabled until approximately 2, after which it leveled out to a more or less constant value, whereas weight showed a steadily decreasing effect with a reduction at higher values.

The robustness of the model was tested by fitting a model containing the predictors considered previously. In the robust model, the effects and statistical significance of all the predictors were confirmed without any major differences. Even the fast-backward selection AIC method did not reject any of the variables considered in the above model.

Therefore, as expected, the multiple logistic regression model showed a significant association between the condition of disability and the factors related to glucose metabolism and insulin resistance, in accordance with the results of the PCA previously presented.

## 5. Discussion

We described a multivariate pattern analysis of metabolic profiles in neurologically impaired children and adolescents. We showed that the variables related to glucose metabolism and insulin resistance were crucial in describing the differences between the group of children with disabilities and the group of healthy control children.

MetS is a constellation of interconnected risk factors of metabolic origin, leading to CVD and diabetes and increased risk of mortality. Due to varying definitions, the prevalence of MetS in the pediatric age remains unclear [33]. In the literature, a median prevalence of 3.3% (range, 0–19.2) in the general population is reported and is significantly higher in overweight (11.9%) and obese (29.2%) subjects [13,14,33].

Although the pathogenetic mechanism of MetS is not fully understood [34,35,36,37], the interaction between obesity, IR, and inflammation is known to play a key role in its development. It is believed that IR initiates different pathogenic pathways, which increase metabolic risk and result in the full expression of MetS [35,38]. IR leads to a decrease in its effect on the suppression of hepatic glucose production in the liver [33]; additionally, hyperinsulinemia leads to increased TG production through an increase in transcription of hepatic lipogenic genes. The increase in free fatty acid delivery results in hepatic insensitivity to the inhibitory effects of insulin on very-low-density lipoprotein (VLDL) secretion and overproduction of triglyceride-rich VLDL particles [33]. Elevated BP in MetS is considered to be related to IR via mechanisms such as sympathetic nervous system activity, renal sodium retention, and smooth muscle growth [33]. IR also influences endothelial dysfunction and altered vasodilatory response [33].

Recently, an increased prevalence of MetS (more than 10%) has also been described in children and adolescents with neurological impairments [12,15,20,21,31]. In this population, IR was reported in 50% of patients, and the alteration in their metabolic profile was not related to ponderal excess. On the contrary, MetS seems to be relevant in malnourished patients [15]. In these patients, several factors including the physiologic adaptive mechanism for human survival [15,17,18,19,20,21], restricted physical activity, chronic stress, altered body composition, and long-term therapy may alter the anabolic/catabolic hormonal balance, leading to glucose, lipid, and insulin signaling interference [15,17,18,19,20,21].

In this report, we re-examined the metabolic profile from different points of view. Applying univariate tests to individual response variables has the advantage of simplicity of interpretation. On the other hand, it fails to account for the correlation in the data. In contrast, multivariate statistical techniques might more adequately capture the multi-dimensional metabolic pattern of health-related conditions. In our study, PCA results and biplots of Figure 2 provide a direct view of the association among the relevant variables, which can be grouped accordingly. These kinds of results are not available from basic univariable analyses and tabulations. Multivariable analysis techniques should be used extensively and consistently in pediatrics to allow results to be compared in a more robust way and to exploit the study of multivariate association structures.

We noted that, compared to the healthy controls, the children-with-disabilities group showed a more dispersed distribution of the variables related to glucose metabolism and IR, without significant differences in males and females. Multivariate analysis helps to quickly identify interconnected patterns in unfavorable metabolic profiles for pediatric populations with disabilities; the parameters related to glucose metabolism play a critical role in the difference between the groups. Even though we considered two groups comparable for age, sex, and pubertal status, the influence of pubertal timing on the impact of IR could not be excluded in children with disabilities [39]. The results confirm that IR is a precocious metabolic alteration that could influence the severity of metabolic involvement. The contribution of IR to the progression of skeletal muscle wasting and lipolysis should also be considered [40]. Screening for the presence of IR should be recommended in subjects with disabilities to prevent the development of complete MetS and the impairment of body composition.

We recognize that our study has some limitations. The number of participants is insufficient for the development of predictive models; in the future, an increased sample size is expected to extend and validate these first results on statistical and clinical grounds. We considered non-obese children with disabilities; the effects on IR, considering HOMA-IR as well as the TyG index, may suggest that muscle or central insulin sensitivity could also be taken into account; further studies on the pathogenic mechanism of IR may be useful in defining the relationship between metabolic parameters. Nutritional status and additional factors, such as familiarity for dysmetabolic diseases, and level of physical activity, could additionally be included in the analysis to better evaluate the causal links between variables.

## 6. Conclusions

A different metabolic profile is found in children and adolescents with disabilities compared to controls. MetS is a crucial point to consider in the treatment and care of this fragile pediatric population. Careful evaluation, periodic monitoring, and measuring of the metabolic state are recommended in these children in order to better define their needs and decrease the risk of morbidity and mortality. Early detection of the interrelated variables and intervention on these modifiable risk factors for metabolic disturbances play a central role in pediatric health and life expectancy in patients with a severe disability.

## Figures and Tables

**Table 1:** Clinical features of the patients and controls and variables included in the analysis.

Variables	Children with Disabilities *n* = 44	Healthy Control Children *n* = 120
Age * (years)	14.47 (6.85, 17.62)	11.01 (8.87, 13.32)
Tanner stages (n)		
-Tanner 1	15 (34.1%)	36 (30.0%)
-Tanner 2–3	23 (52.3%)	66 (55.0%)
-Tanner 4–5	6 (13.6%)	18 (15.0%)
Height * (cm)	135.5 (114.2, 145.2)	143.9 (135.3, 154.1)
Height z-score [32]	−2.01 (−4.02, −0.76)	−0.14 (−0.87, 0.98)
Weight * (kg)	27.55 (17.68, 34.70)	35.50 (30.00, 46.25)
Weight z-score [32]	−0.67 (−1.52, 0.27)	0.76 (−0.47, 1.37)
Body mass index (kg/m^2^) *	15.70 (13.82, 18.05)	17.35 (15.79, 19.53)
BMI z-score [32]	−1.36 (−2.95, −0.12)	0.076 (−0.53, 0.77)
Fasting glucose (mg/dL) *	73.00 (59.75, 90.00)	75.00 (69.00, 80.00)
Fasting insulin (U/mL) *	14.95 (5.55, 23.07)	5.300 (3.100, 8.40)
HOMA-IR *	3.01 (1.02, 4.64)	0.58 (0.31, 1.12)
Fasting triglycerides (mg/dL) *	83.5 (66.0, 106.5)	46.0 (38.0, 57.0)
Total cholesterol (mg/dL) *	134.5 (110.8, 166.2)	156.0 (136.0, 170.8)
HDL cholesterol (mg/dL) *	43.00 (37.00, 48.00)	53.50 (47.00, 60.00)
Systolic blood pressure (mmHg) *	100.5 (90.5, 116.0)	100.0 (95.0, 110.0)
Diastolic blood pressure (mmHg) *	65.00 (56.00, 76.00)	60.00 (60.00, 70.00)
Trygliceride glucose index *	8.08 (7.80, 8.35)	7.44 (7.22, 7.67)

* Data are expressed as median and 25% and 75% quantiles. HOMA-IR= homeostasis model assessment for insulin resistance.

## Data Availability

Data are contained within the article.
